# Mapping system for portal placement in laparoscopic procedures of small animals

**DOI:** 10.1186/s12917-015-0524-4

**Published:** 2015-08-16

**Authors:** Nikola Katic, Vivian Fromme, Barbara Bockstahler, Gilles Dupré

**Affiliations:** Small Animal Surgery, Department for Companion Animals and Horses, University of Veterinary Medicine, Veterinaerplatz 1, 1210 Vienna, Austria; Present address: Universität Leipzig, Veterinärmedizinische Fakultät, Klinik für Kleintiere, An den Tierkliniken 23, 04103 Leipzig, Germany

**Keywords:** Laparoscopy, Portal placement, Minimally invasive surgery

## Abstract

**Background:**

Current recommendations for portal placement in laparoscopy are often imprecise. The aim of this study was to establish and evaluate a mapping system for portal placement during laparoscopic procedures in small animals.

Sixty-four final-year veterinary students took part in this *in papyro* study.

Descriptions of portal placements of two recent veterinary laparoscopic papers were randomly chosen as templates. The students performed portal placement based either on the description in the papers or based on the orthogonal mapping system for portal placement developed by the authors in a previous pilot study. The participants were randomly divided into two groups and asked to virtually chart positions of the portals on two photographs of a dog’s abdomen. Group A (*n* = 31) placed the portals using the mapping system, and Group B (*n* = 33) placed the portals based on the explanations provided in two randomly selected studies.

**Results:**

Group A achieved an overall correct placement rate of 94.91 % (87.1–100.0 %) with an overall mean distance of 1.31 mm (0.00–3.61 mm) from the desired placement points. Group B achieved an overall correct placement rate of 40.8 % (3.1–93.3 %) with an overall mean distance of 16.97 mm (7.17–27.63 mm) from the desired placement points. The students in Group A performed significantly better than did students in Group B (*P* < .05).

**Conclusions:**

Use of the mapping system significantly improved correct portal placement in a dog photograph model. Use of such systems in laparoscopy may help facilitate correct portal placement and improve the repeatability of procedures, especially for the novice surgeon.

## Background

The first minimally invasive laparoscopic approach to the abdominal organs in a dog was experimentally performed at the beginning of the twentieth century [1]. In 1910, Jacobeus successfully performed the first diagnostic laparoscopy procedures in humans. He described the risk of injuring the abdominal organs while placing the trocars and recommended practical training on animals and corpses prior to the performance of laparoscopic procedures in human patients [2]. Since then, although portal placement for the laparoscope and various instruments is considered to be essential, very little consensus exists on the optimal portal placement for a given procedure [3, 4]. Moreover, even in the most frequently cited studies and validated techniques, instructions regarding the location of portal placement remain imprecise [3, 5]. In publications where instructions are given in metric units [4, 6, 7] different sizes of animal patients are often not accounted for and such instructions might not be applicable for different populations of patients. The lack of such information may lead to surgical error, especially in the hands of novice surgeons.

The goal of this study was to compare the accuracy of laparoscopic portal placement among two groups of veterinary students - one group using established publication guidelines, the other using the authors’ orthogonal mapping system. Our hypothesis was that portal placement using the mapping system would be more accurate and reproducible than that following previously published methods.

## Methods

### Students

Sixty-four final-year veterinary students were enrolled in this study after having verbally agreed to participate. All students in both groups had achieved the same level of education and were given the same amount of time to fulfill the tasks. They were randomly divided into 2 groups by means of a lottery drawing. Group A (*n* = 31) placed the portals using the mapping system, and Group B (*n* = 33) placed the portals based on the explanations provided in two studies [3, 7]. These two studies were discretionarily selected by the authors based on different levels of complexity of portal placement: a simpler procedure with 3 entry points, 2 of which were symmetrical [3], and a more complex procedure with 4 entry points, none of which were symmetrical [7].

### Ethical approval

A verbal consent was obtained from the students who participated in this study. They were offered to voluntarily participate or decline participation in this study. An official ethical approval was not obtained as the authors were not aware of this requirement by the time this study was conducted.

### Mapping system for portal placement

The mapping system incoroprates an orthogonal Cartesian coordinate system with both an x- and y-axis. For the purpose of this study, the coordinate system was plotted over a photograph of the abdomen of a dog in dorsal recumbency. The umbilicus was set as the center (0, 0), the y-axis connected the umbilicus to the xiphoid, and the x-axis was perpendicular to the y-axis at the level of the umbilicus. One-fourth of the distance between the umbilicus and xiphoid was defined as the basic unit. One-fourth of the basic unit was defined as a subunit.

The location of the portal (or point on the coordinate system) was defined in relation to the center of the system. Therefore, portal placement in this study was performed in two defining steps: first, the point was defined in larger basic units; second, the point was more finely defined in smaller subunits. In this way, every point was defined as (X, x) and (Y, y), where X and Y represent basic units and x and y represent subunits. The point was further defined with the use of either ‘+’ (to the right and upward from 0) or ‘–’ (to the left and downward from 0). In this mapping system, the basic unit was 2.0 cm and the subunit was 0.5 cm.

### Published guidelines for portal placement

Descriptions of portal placements were extracted from two previously published articles and translated into the students’ native language. Procedure 1 in the present study was based on the following description of laparoscopic ovariohysterectomy [3]: For the 1st portal, ‘a 1-cm, skin incision was made over the umbilicus, exposing the linea alba.’ For the 2nd and 3rd portal: ‘two skin incisions were made in a nonvascular area, paramedian to the midline, and at the level of the inguinal fold.’ Procedure 2 in the present study was based on the following description of laparoscopic cholecystectomy [7]: ‘A 4 portal technique was used with the 1st portal… established 1 cm caudal to the umbilicus… three instrument portals were established… under direct observation: one 5 cm lateral, and 3 cm cranial to the umbilicus on the left side, and 2 located 3 and 5 cm lateral to the umbilicus on the right side.’ In this particular article the values were given as ranges (e.g., 5–8 cm), however for the sake of comparability this was adapted so that the lower number in the range was used (e.g., 5 cm instead of 5–8 cm). Text passages, which were irrelevant for portal placement, were omitted.

### Virtual models

A virtual model was created by printing a photograph of a dog (medium size, female, mongrel; 22 kg) in dorsal recumbency for each of the students to use for portal placement. When distances were given as a range, the shortest distance was used. Students in Group A plotted the portal holes using the mapping system, while students in Group B placed the portals based on the explanations cited in the two selected studies.

**Fig. 1:**
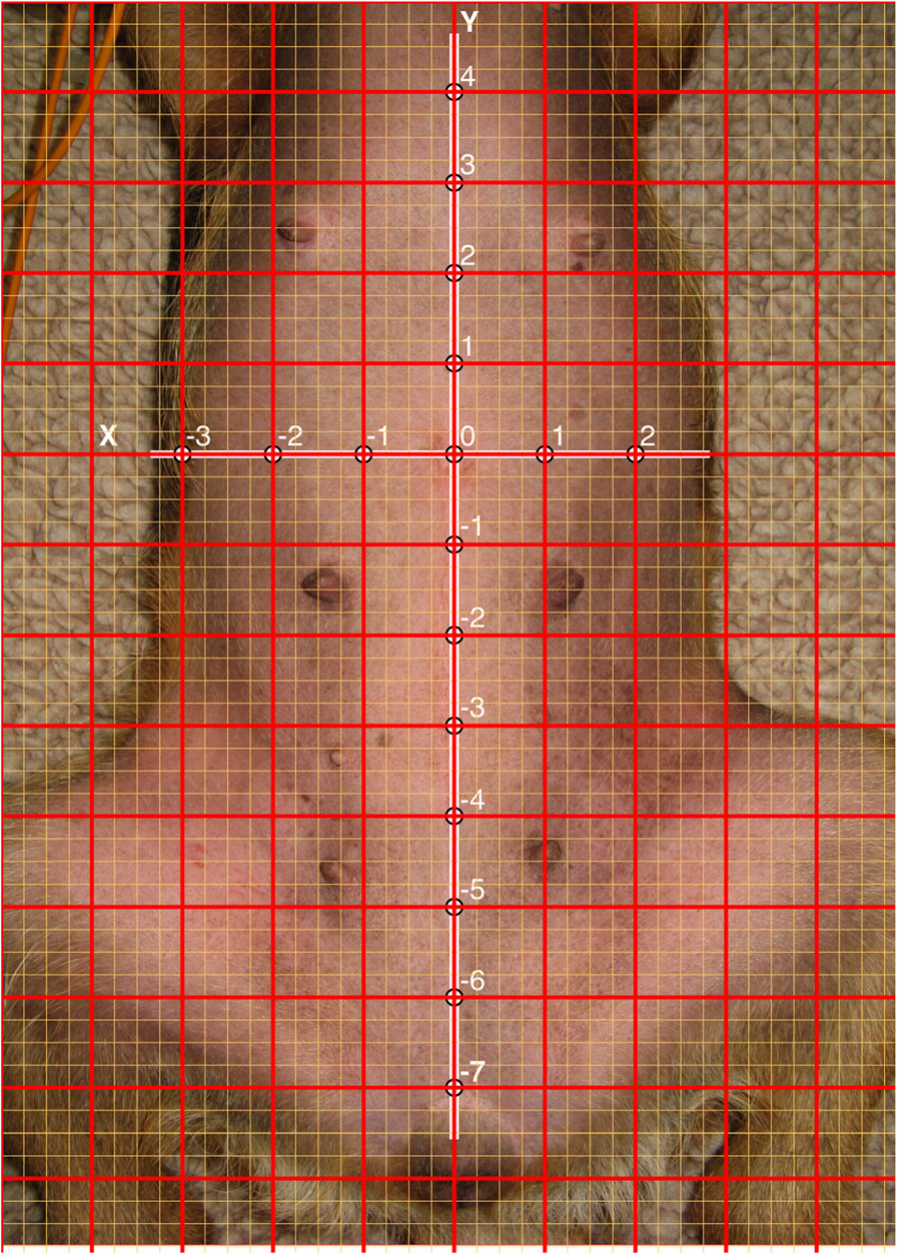
Photograph of the abdomen of a dog in dorsal recumbency with the mapping system shown centered at the umbilicus

The model for Group A is shown in Fig. 1. The portal placement descriptions in the above-mentioned studies were translated into the mapping system. The students placed ‘portals’ by marking crosses at the following locations:

$$ \begin{array}{c}\hfill \mathrm{Procedure}\kern0.49em 1:\kern0.37em 1.\ \mathrm{X}\left(0,\ 0\right),\ \mathrm{Y}\ \left(0,\ 0\right)\hfill \\ {}\hfill \kern4.32em 2.\kern0.37em \mathrm{X}\;\left(-2,+1\right),\ \mathrm{Y}\left(-4,+1\right)\hfill \\ {}\hfill \kern4.32em 3.\ \mathrm{X}\;\left(+2,-1\right),\ \mathrm{Y}\left(-4,+1\right)\hfill \end{array} $$

$$ \begin{array}{c}\hfill \mathrm{Procedure}\ 2:\ 1.\ \mathrm{X}\ \left(0,\ 0\right),\ \mathrm{Y}\left(-1,+2\right)\hfill \\ {}\hfill \kern3.96em 2.\ \mathrm{X}\left(+2,+2\right),\mathrm{Y}\left(+1,+2\right)\hfill \\ {}\hfill \kern3.24em 3.\ \mathrm{X}\;\left(-1,-2\right),\ \mathrm{Y}\left(0,0\right)\hfill \\ {}\hfill \kern3.24em 4.\ \mathrm{X}\;\left(-2,-2\right),\ \mathrm{Y}\left(0,0\right)\hfill \end{array} $$

**Fig. 2:**
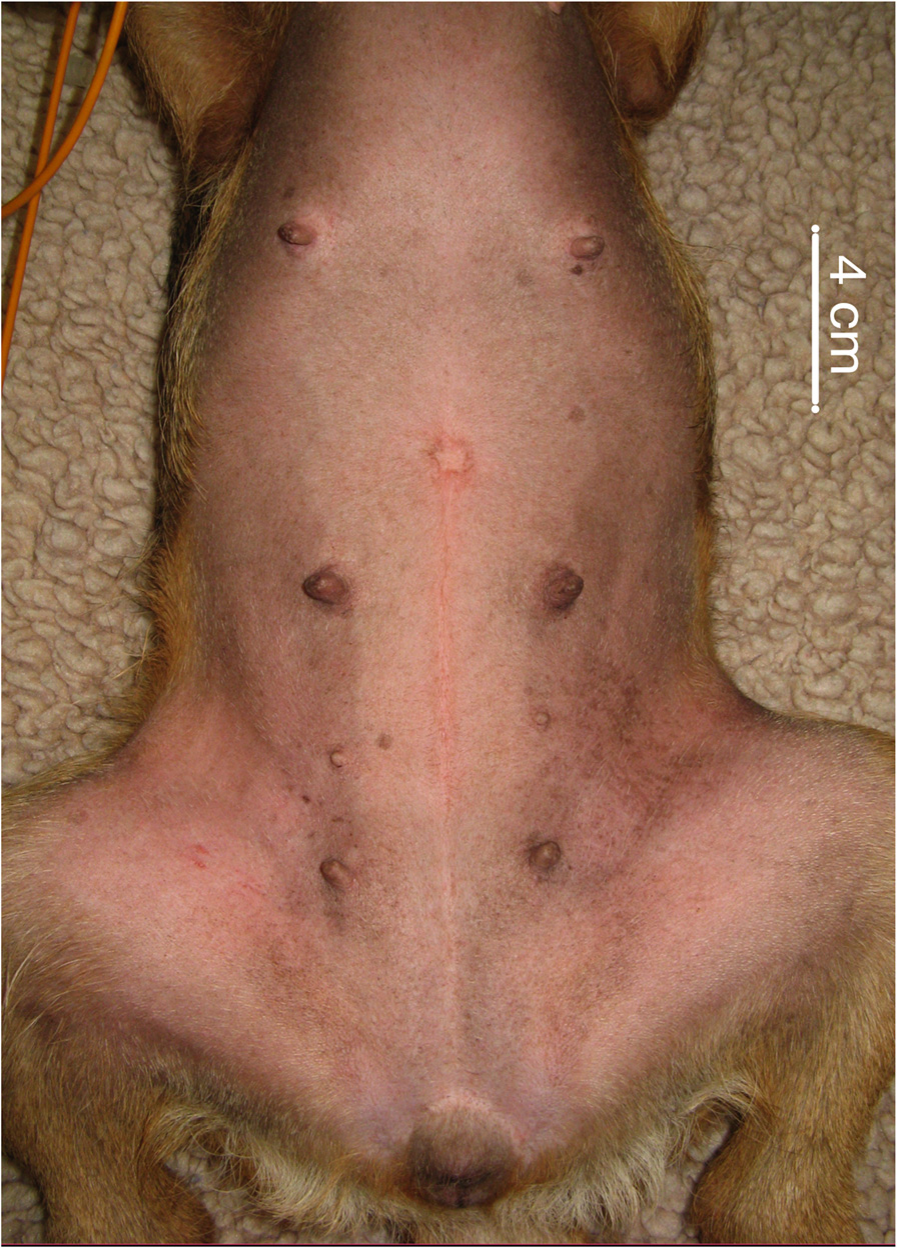
Photograph of the abdomen of a dog in dorsal recumbency without the mapping system shown. Note the 4 cm distance guide included on this photograph as a measurement reference during portal placement

The model for Group B is shown in Fig. 2. The students in Group B used the same photograph as that used in Group A, but without the mapping system. A 4-cm-long distancer was plotted to help with orientation. These students placed ‘portals’ by marking crosses according to the descriptions provided in the two reference studies.

### Assessment of the groups

The performance of students in both groups was assessed using two transparent templates with already-marked virtual portal placement points positioned over the photographs (Figs. 1, 2). The points were centered on the umbilicus and had the same dimensions as the coordinate system used for Group A. All virtually placed points were verified against the templates, and the distances between them and the correct points were measured with a millimeter ruler. Virtual placement within a distance of 10 mm was considered to be correct.

### Statistical analysis

Statistical analysis of the results was performed by PASW/SPSS Statistics for Windows (Version 17.0; SPSS, Inc., Chicago, IL). Descriptive statistics were performed. A *t*-test for independent samples was used to compare the mean distance from the correct placement between the 2 groups. A *P* value of < 0. 05 was considered to be statistically significant.

## Results

All placements within 10 mm of the templates were evaluated. The numbers and percentage of correct portal placement were determined (Table 1). The mean distance from the desired placements was provided in millimeters, and comparison of each placement between the two groups was performed using an independent-samples *t*-test (Table 2). Overall 10 out of 231 of the portal placements in Group B were excluded from the analysis because of the use of several-millimeter circles instead of crosses, making evaluation of the true distance impossible. All incorrect markings occurred within Group B (1–3 per placement). Table 1 includes the numbers of portal placements used in the study.

**Table 1:** Numbers and rates of correct portal placement within 10 mm of template and number of placements used in the group B after exclusion of incorrect ones

Placement	Group A (*n* = 31)	Group B	Number of placements used in group B
Placement 1, Procedure 1	31/100.0 %	17/54.8 %	*n* = 31
Placement 2, Procedure 1	31/100.0 %	1/3.1 %	*n* = 32
Placement 3, Procedure 1	31/100.0 %	1/3.1 %	*n* = 32
Placement 1, Procedure 2	27/87.1 %	28/93.3 %	*n* = 30
Placement 2, Procedure 2	29/93.5 %	4/12.5 %	*n* = 32
Placement 3, Procedure 2	29/93.5 %	23/71.9 %	*n* = 32
Placement 4, Procedure 2	28/90.3 %	15/46.9 %	*n* = 32

**Table 2:** Comparison of portal placement between the two groups

Placement	Group	Mean distance from desired placement (mm)	Standard deviation	*P* value
Placement 1, Procedure 1	A	0.000	0.000	.000
	B	9.550	9.922	
Placement 2, Procedure 1	A	0.000	0.000	.000
	B	27.630	12.037	
Placement 3, Procedure 1	A	0.000	0.000	.000
	B	27.500	9.415	
Placement 1, Procedure 2	A	1.900	5.029	.022
	B	7.170	11.384	
Placement 2, Procedure 2	A	2.160	6.378	.000
	B	22.720	16.555	
Placement 3, Procedure 2	A	1.480	6.104	.002
	B	10.560	13.988	
Placement 4, Procedure 2	A	3.610	13.328	.004
	B	13.660	13.656	

Students in Group A exhibited significantly improved performance for all placements. For Procedure 1 in Group A, 100 % of the placements were correct for all 3 points with no deviation. For students in Group B, the first placement was correct in 54.8 % of cases with a mean distance of 9.55 mm from the desired point. The other 2 placements were correct in only 3.1 % of cases with a mean distance of 27.63 and 27.50 mm for placements 2 and 3, respectively. A statistically significant difference was observed among all 3 points between the 2 groups (*P* = .000).

For Procedure 2 in Group A, portal placements were correct in 87.1 %, 93.5 %, 93.5 %, and 90.3 % of cases with a mean distance of 1.90, 2.16, 1.48, and 3.61 mm for placements 1, 2, 3, and 4, respectively. Group B achieved correct portal placement in 93.3 %, 12,5 %, 71,9 % and 46.9 % of cases with a mean distance of 7.17, 22.72, 10.56, and 13.66 mm for placements 1, 2, 3, and 4, respectively. It should be noted that group B had a slightly better percentage of correct placements for the first point than did Group A (93.3 % versus 87.1 %, respectively), but exhibited a greater mean distance (7.17 mm).

However, the overall performance of Group A for placement 1 was significantly better than that of Group B (*P* = .022). A statistically significant difference was observed among all 4 points between the 2 groups (*P* < .05).

Group A achieved an overall correct placement rate of 94.91 % (87.1–100.0 %) with an overall mean distance of 1.31 mm (0.00–3.61 mm) from the desired placement points. Group B achieved an overall correct placement rate of 40.8 % (3.1–93.3 %) with an overall mean distance of 16.97 mm (7.17–27.63 mm) from the desired placement points. Overall the students in Group A performed significantly better than did those in Group B (*P* < .05).

## Discussion

In this study, the students who used the mapping system (Group A) exhibited a significantly greater percentage of correct placements than did the students who used the written descriptions (Group B). Moreover, when the entry point was misplaced, the mean distance to the desired point was significantly smaller in Group A than in Group B. Accessing the abdominal cavity during laparoscopic procedures can be challenging. In humans, more than 50 % of injuries to the gastrointestinal tract and major blood vessels in such procedures occur at the very beginning, as the portals are being placed [8]. Correct portal placement facilitates direct access to the target organs while providing adequate visualization of the surgical field and anatomic surroundings [9]. Although several studies have evaluated and reviewed the safety of laparoscopic instrument entry into the abdominal cavity, [9–11] surgeons’ experience and medical intuition remain the most important guidelines for primary and secondary portal placement. The improved performance of the students who used the mapping system in this study suggests the value of a systematic approach for reproducible trocar placement, especially for surgeons with no or minimal experience with laparoscopic surgery.

As laparoscopy continues to gain popularity in veterinary medicine, the various laparoscopic procedures that are performed increase in complexity. A wide variety of procedures have been performed in small animals and range from simpler operations such as laparoscopic biopsy [12] and castration [3, 6] to more complex ones such as laparoscopic adrenalectomy [5] and cholecystectomy [7]. To evaluate our sample population, we selected two procedures with different levels of complexity: a simpler procedure with 3 entry points, 2 of which were symmetrical [3], and a more complex procedure with 4 entry points, none of which were symmetrical [7]. How the higher complexity of the entry points in the second procedure influenced our results remains unclear.

In this mapping system, the same coordinate can be defined in 2 different ways: by either choosing the smaller unit and adding the subunits or choosing the greater unit and reducing it by the subunits. For the first entry point in the second procedure, we defined the Y coordinate as (−1, +2). The other possibility of expressing the Y coordinate is (0, −2). Whether this would have improved the Group A students’ performance of correct placement of the first entry point in the second procedure is unclear. Group B exhibited a slightly better performance for this same entry point; correct virtual placement was achieved in 93.3 % of the students (versus 87.1 % of the students in Group A). However, the misplacement distance of this point was significantly greater in Group B than in Group A (7.17 ± 11.38 versus 1.90 ± 5.03 mm, respectively; *P* < .05). The clinical significance of these findings with respect to this particular entry point cannot be derived from our study.

Different mapping systems were previously tested in a pilot study conducted by the authors (unpublished data). The herein-described Cartesian (orthogonal) coordinate system [13] was chosen due to its simplicity and well-known applicability in the natural sciences. The main advantage of this system is its ability to uniquely define a point in a plane by 2 numerical coordinates. A simplified version of this system, namely the 4-abdominal-quadrant descriptive system with the center at the umbilicus, is routinely used in the clinical setting [14]. The umbilicus was also chosen to be the center of our system. One-fourth of the distance between the xiphoid and umbilicus was chosen as the basic unit, and one-fourth of the basic unit was chosen as a subunit to facilitate fine adjustment of the placement of entry points. We selected the umbilicus and the xiphoid because they are easily accessible under clinical application of the system. A potential advantage of the mapping system over descriptive trocar placement is that the size of the units and subunits changes with the distance between the xiphoid and umbilicus or with the size of the patient. This is not the case in descriptive explanations, where distances are often given in metric units [4, 6, 7]. The same distance (e.g., 2 cm caudal to the umbilicus) is not located at the same anatomical site among patients of different size. Several studies have assessed the anatomical location of the umbilicus in humans [15–17]. In normal, healthy, nulliparous humans, the navel seems to have a constant anatomical position. The xiphoid–navel: navel–cranial pubic symphysis ratio can be approximated at 55:45 in humans. However, such precise relationships in animal patients are unknown and warrant further assessment. Since this study was performed on a single virtual model, which was the same for all participants, any variation of the navel–xiphoid distance could be neglected.

Despite our promising results, important limitations of our study should be noted. First, because of the use of a virtual model, definitive conclusions on the use of this system under clinical conditions are lacking. Second, testing was performed in a 2-dimensional virtual environment, and the influences of abdominal shape on trocar placement were not evaluated. Third, we chose inexperienced veterinary students to compare portal placement, and our results likely would have been different with more experienced participants.

## Conclusions

In conclusion, the use of the described mapping system applied in a virtual small animal model improved the accuracy of portal placement by veterinary students who had minimal experience in laparoscopic procedures. The authors hope that this report will stimulate interest and discussion within the laparoscopic community. Further studies of the clinical applicability of mapping systems for laparoscopic portal placement in animals and humans are warranted.
